# 应用生物发光技术研究甲基硒酸对L9981-Luc肺癌细胞株移植瘤模型生长转移的影响

**DOI:** 10.3779/j.issn.1009-3419.2013.02.02

**Published:** 2013-02-20

**Authors:** 苑蓉 任, 玉丽 王, 红雨 刘, 惠琴 闫, 军 陈, 梅 侯, 为民 李, 亚光 范, 清华 周

**Affiliations:** 1 610041 成都，四川大学华西医院 West China Hospital, Sichuan University, Chengdu 610041, China; 2 300052 天津，天津市肺癌转移与肿瘤微环境重点实验室，天津市肺癌研究所，天津医科大学总医院 Tianjin Key Laboratory of Lung Cancer Metastasis and Tumor Microenvironment, Tianjin Lung Cancer Institute, Tianjin Medical University General Hospital, Tianjin 300052, China

**Keywords:** 肺肿瘤, 甲基硒酸, 生物发光成像, Lung neoplasms, Methylseleninic acid, Bioluminescence imaging

## Abstract

**背景与目的:**

甲基硒酸是一种新型的人工合成的硒化合物。研究发现甲基硒酸对肿瘤细胞的生长转移有抑制作用。本研究的目的是探讨甲基硒酸对L9981-Luc裸鼠异体移植瘤生长和转移能力的抑制作用及机制。

**方法:**

建立L9981-Luc肺癌细胞株移植瘤模型，用精诺真活体动物可见光成像系统观察肺癌移植瘤肿瘤生长转移情况。实验将6周龄裸鼠15只，随机分为3组，每组5只，对照组每日腹腔注射生理盐水0.2 mL；甲基硒酸组每日腹腔注射甲基硒酸溶液50 μg（0.2 mL）；顺铂组每周腹腔注射顺铂4 mg/kg。

**结果:**

接种第21天，不同组间原发瘤发光值比较差异有统计学意义（*P*=0.002）；顺铂组发光值明显低于对照组（*P*=0.001），甲基硒酸组发光值明显低于对照组（*P*=0.031）。不同药物处理组胸部转移信号发光值差异无统计学意义（*P* > 0.05）。

**结论:**

甲基硒酸能明显抑制L9981-Luc裸鼠异体移植瘤生长，并有抑制L9981-Luc原发瘤肺转移的趋势。

## Abstract (en)

**Background and objective:**

Methylseleninic acid (MSA) is an artificially developed selenium compound. It has been proven that MSA could inhibit growth and metastasis on many tumor cells. This study investigated whether MSA has an impact on the growth and metastasis of L9981-Luc lung cancer transplanted model in nude mice or not.

**Methods:**

A transplantated tumor model was established in nude mice. Fifteen nude mice were randomly divided into three groups: the control group treated with normal saline (0.2 mL/d), the MSA group treated with MSA solution (0.2 mL), and the cisplatin (DDP) group injected intraperitoneally with DDP (4 mg/kg/w). Inhibition of MSA on tumor growth and tumor metastasis was observed using the IVIS Imaging System 200 Series.

**Results:**

A significant difference was obserced in the primary tumor bioluminescence among the three groups (*P*=0.002) on 21 days post-inoculation. Primary tumor bioluminescence in the DDP group (*P*=0.001) and in the MSA group (*P*=0.031) was significantly lower than that in the control group (*P*=0.001). No significant difference in the metastasis bioluminescence of the thoracic area was indicated among the three groups (*P* > 0.05).

**Conclusion:**

MSA can inhibit the growth of planted tumor of transgenic lung cancer cell lines L9981-Luc in nude mice. MSA may also suppress the distant metastasis of the transplanted tumor of transgenic lung cancer cell lines L9981-Luc in nude mice.

硒作为一种人体必需的微量元素和天然的防癌抑癌制剂，越来越受到肿瘤学界的关注。甲基硒酸（methylseleninic acid, MSA）是一种新型的人工合成的硒化合物，以其广泛的抗癌效应和无需体内代谢酶转化而直接作用于靶细胞的特性成为最具潜力的硒化合物抗癌制剂^[1]^。为进一步探明MSA对肿瘤的抑制作用，本研究应用精诺真活体动物可见光成像系统（IVIS imaging system 200 Series），研究了MSA对肺癌细胞株L9981-Luc裸鼠移植瘤的抑制作用。

## 1 材料与方法

### 1.1 材料

实验细胞株L9981-Luc，由天津市肺癌转移与肿瘤微环境重点实验室提供，已经证明其在体内外均能持续稳定地表达荧光素酶基因，且发光值和细胞数成明显直线相关^[2]^。甲基硒酸由美国加利福尼亚大学Allen C. Gao教授（Department of Urology and Cancer Center, UC Davis School of Medicine, Sacramento, CA）提供。顺铂（DDP）购自齐鲁制药有限公司（批号：030801）。实验动物采用BALB/c裸鼠，购自北京维通利华动物饲养有限公司，雌性，6周龄，质量为18 g-20 g。

Artagain黑纸（Strathmore, Catalog445-109）；注射器、镊子、手术剪；荧光素（Luciferin）购自上海睿星基因技术有限公司；麻醉剂异氟烷（isoflurane）24孔板，Greiner公司产品；RPMI-1640培养基、新生小牛血清购自GIBCO公司；精诺真活体动物可见光成像系统（IVIS imaging system 200 Series）型号：IVIS200，美国Xenogen Corporation产品。

### 1.2 方法

#### 1.2.1 细胞培养和接种

L9981-Luc细胞株在含10%的小牛血清及PRMI-1640培养液中培养，传代扩增，收集对数生长期细胞制备悬液，调整每组细胞浓度为1×10^7^个/mL；台盼蓝染色计数活细胞占95%以上。SPF环境下进行实验操作，每只裸鼠右后腿腹股沟处皮下接种细胞悬液0.1 mL/只。

#### 1.2.2 溶液配制方法

称取10 mg甲基硒酸，加入5 mL生理盐水，配成2 mg/mL甲基硒酸溶液，从其中取2 mL，加入14 mL生理盐水，即配成0.25 mg/mL甲基硒酸溶液；将4 mg/mL的顺铂溶液稀释10倍，即配制成0.4 mg/mL的顺铂溶液。

#### 1.2.3 实验分组

6周龄裸鼠，体重约16 g-18 g，共15只，随机分为3组，每组5只，接种第3天开始给药：对照组每日腹腔注射生理盐水0.2 mL，连续14天；甲基硒酸组每日每只腹腔注射甲基硒酸溶液50 μg（0.2 mL），连续14天；顺铂组每周每只腹腔注射顺铂4 mg/kg。

#### 1.2.4 抑瘤率的计算

$
			 抑瘤率\left( \%  \right) = \frac{{对照组瘤发光值-实验组瘤发光值}}{{对照组瘤发光值}} \times 100\% 
			 $

### 1.3 统计学处理

采用SPSS 11.0统计软件。本组实验数据为计量资料，采用方差分析，以*P*＜0.05为差异有统计学意义。

## 2 结果

### 2.1 不同药物处理组不同时间L9981-Luc原位瘤发光强度的比较（图 1）

**1:**
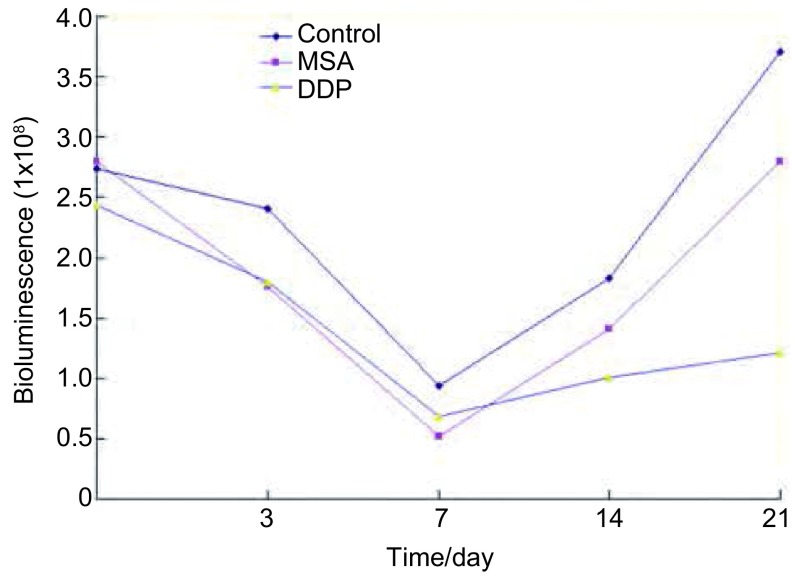
不同药物处理组和不同时间组间人高转移大细胞肺癌细胞株L9981-Luc原位移植瘤发光强度比较 Comparison of planted tumor growth of L9981-Luc treated with different drugs in nude mice

皮下接种L9981-Luc肺癌细胞株的荧光信号在接种后逐渐增强。接种后第7天，所有裸鼠接种部位皮下均可探测到1×10^5^-1×10^6^数量级的信号强度，随时间增加，原发瘤和胸部转移信号均同步增加（图 2）。接种后第0-14天，不同组人高转移大细胞肺癌细胞株L9981-Luc移植瘤在裸鼠体内发光强度无统计学差异（表 1）；用药第21天，不同组间移植瘤发光值比较差异有统计学意义（*P*=0.002）（表 2）；两两比较：对照组发光值显著高于顺铂组（*P*=0.001），对照组发光值显著高于甲基硒酸组（*P*=0.031），甲基硒酸和顺铂组间比较，差异无统计学意义（*P*=0.147）。MSA组抑瘤率为25.20%，DDP组67.29%（图 3）。

**2:**
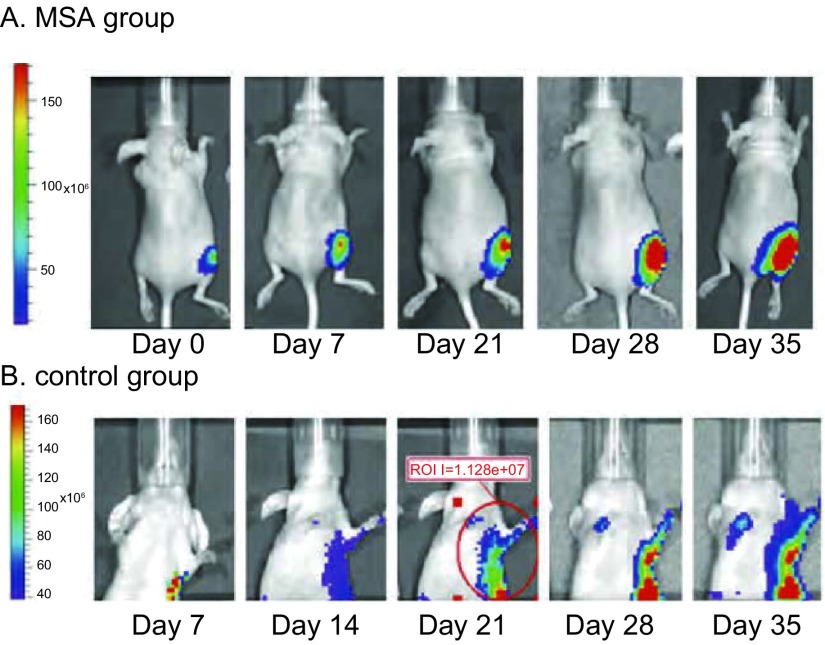
与MSA组比较，对照组活体内移植瘤和转移瘤信号值随时间递进而逐渐增强 Compared with MSA group, bioluminescence of primary tumor and metastases in the thoracic area in the control group was gradually increased as time increased

**1:** 0-14天不同药物处理组L9981-Luc原位瘤发光强度的比较（Mean±SD） Comparison of the bioluminescence (p/s) of primary tumor of L9981-Luc treated with different drugs d0-d14 (Mean±SD)

Time	Control	MSA	DDP	*P*
Day 0	2.741×10^8^±2.744×10^7^	2.795×10^8^±3.335×10^7^	2.436×10^8^±4.412×10^7^	0.545
Day 3	2.410×10^8^±1.357×10^7^	1.759×10^8^±2.159×10^7^	1.801×10^8^±3.465×10^7^	0.592
Day 7	9.427×10^7^±2.972×10^7^	5.215×10^7^±7.749×10^6^	6.879×10^7^±1.734×10^7^	0.593
Day 14	1.824×10^8^±2.828×10^7^	1.410×10^8^±3.615×10^7^	1.005×10^8^±2.092×10^7^	0.073
MSA: methylseleninic acid; DDP: cisplatin.				

**2:** 第21天不同药物处理组L9981-Luc原位瘤发光强度的比较（Mean±SD） Comparison of the primary tumor bioluminescence (p/s) of L9981-Luc treated with different drugs on d21 (Mean±SD)

Time	Control	MSA	DDP	*P*
Day 21	3.702×10^8^±3.104×10^7^	2.769×10^8^±3.313×10^7^	1.211×10^8^±2.390×10^7^	0.002
Inhibitory rate (%)	0	25.20%	67.29%	-

**3:**
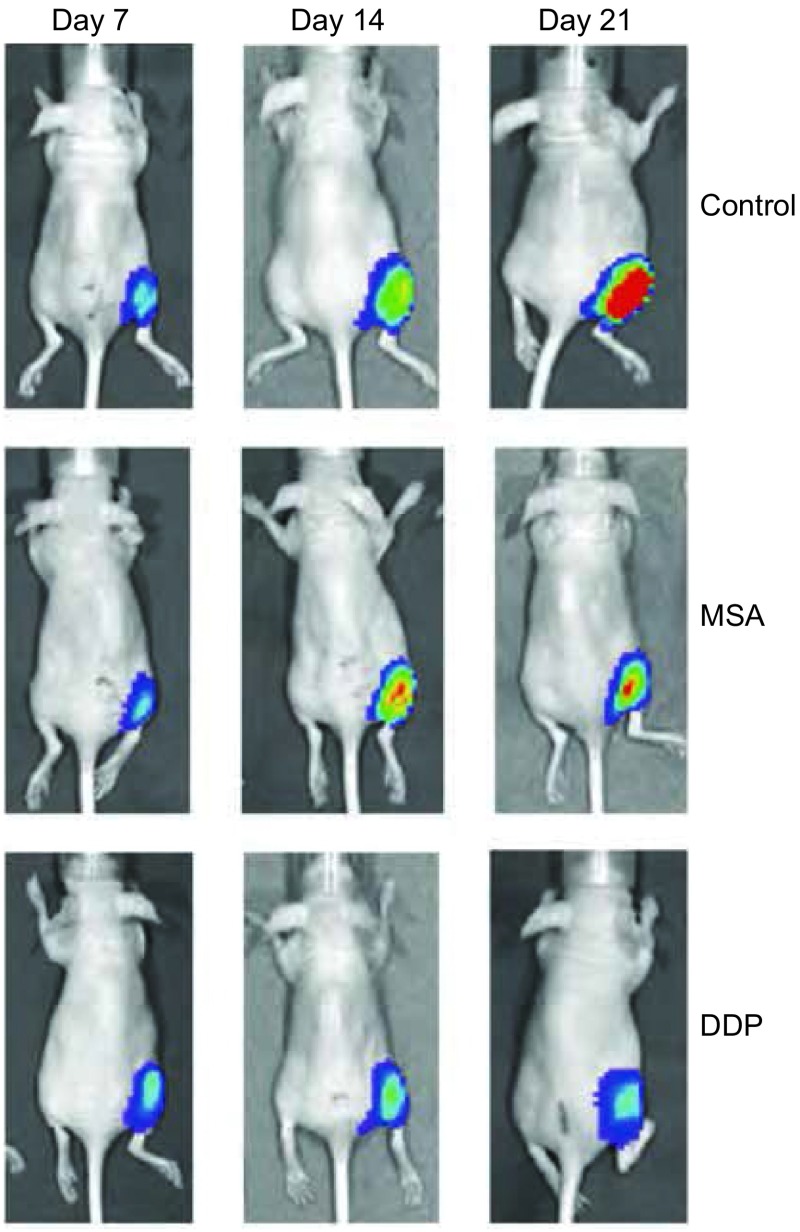
分别在第7、14、21天测量各药物处理组各只裸鼠移植瘤发光信号值，计算平均值，两两比较，对照组明显高于顺铂和甲基硒酸组（*P*＜0.001)，甲基硒酸组明显高于顺铂组（第21天，*P*＜0.05）。 Measure the mean bioluminescence (p/s) of primary planted tumor on d7, d14, d21. The mean bioluminescence of the planted tumor in control group was remarkably higher than that in the DDP group and MSA group (*P* < 0.001), MSA group was remarkably higher than that in control group on d21 (*P* < 0.05).

### 2.2 不同药物处理组间L9981-Luc移植瘤肺转移情况的比较

于接种后第7天开始观察到不同药物处理组胸部转移信号，发光值随时间逐渐增强，分别在d7、d14、d21测量各药物处理组各只裸鼠胸部转移信号值（图 4），计算平均值，对照组发光值明显高于顺铂和甲基硒酸组，甲基硒酸组高于顺铂组（*P*＞0.05）。MSA组抑瘤率为17.14%，DDP组为48.30%（表 3，图 5）。

**4:**
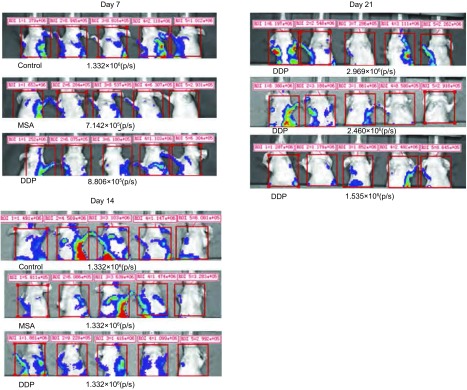
别在第7、14、21天测量各药物处理组裸鼠胸部转移信号值，计算平均发光值，对照组明显高于顺铂和甲基硒酸组（*P*＜0.001），甲基硒酸组高于顺铂组（*P*＞0.05）。 Measure the mean bioluminescence (p/s) of metastases in the thoracic area on d7, d14, d21. The mean bioluminescence of the metastases in control group was remarkably higher than that in the DDP group and MSA group (*P* < 0.001), MSA group was remarkably higher than that in control group (*P* > 0.05).

**3:** 不同药物处理组间L9981-Luc胸部转移信号发光强度的比较（Mean±SD） Comparison of the mean bioluminescence (p/s) of thoracic area metastases of L9981-Luc treated with different drugs over time (Mean±SD)

Time	Control (p/s)	MSA (p/s)	DDP (p/s)	*P*
Day 7	1.332×10^6^±1.598×10^5^	7.142×10^5^±8.570×10^4^	8.806×10^5^±2.057×10^5^	0.001
Day 14	2.186×10^6^±2.618×10^5^	1.917×10^6^±2.300×10^5^	1.123×10^6^±1.948×10^5^	0.031
Day 21	2.969×10^6^±3.563×10^5^	2.460×10^6^±2.952×10^5^	1.535×10^6^±1.842×10^5^	0.040
Inhibitory rate (%)	0	17.14%	48.30%	-

**5:**
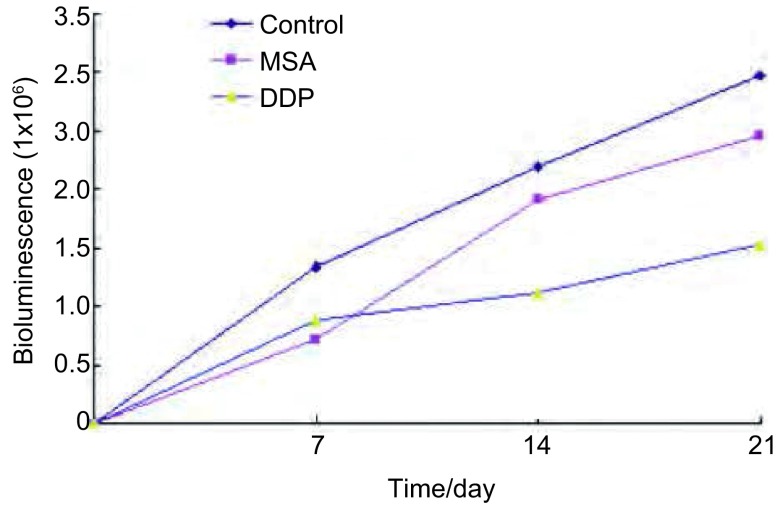
不同药物处理组和不同时间人高转移大细胞肺癌细胞株L9981-Luc在裸鼠胸部转移瘤生长的组间比较 Comparison of metastatic tumor growth of L9981-Luc planted tumor treated with different drugs in nude mice

### 2.3 各组裸鼠体重改变的比较

对照组裸鼠体重平均增加2.10 g，体重增加率为12.6%，甲基硒酸组平均体重增加2.10 g，体重增加率为11.9%，顺铂组平均体重变化为-0.64 g，变化率为-3.59%，不同药物处理组间裸鼠体重增加差异有统计学意义（*P*=0.038）；两两比较，对照组裸鼠体重增加值明显高于顺铂组（*P*=0.029），甲基硒酸组裸鼠体重增加值明显高于顺铂组（*P*=0.034）（表 4）。

**4:** 用药前后各组小鼠体重变化情况（Mean±SD） The weight change before and after drug use (Mean±SD)

Item	Control	MSA	DDP	*P*
Body weight before treatment (g)	16.68±0.79	17.64±0.64	17.82±0.50	0.030
Body weight after treatment (g)	18.78±2.33	19.74±1.68	17.18±0.91	0.104
Increase of body weight (g)	2.10±1.89	2.10±1.39	-0.64±0.94	0.038
Rate (%)	12.6%	11.9%	-3.59%	-

## 3 讨论

本研究将带有荧光素酶基因（*Luc*）的质粒PGL4.17经阳离子脂质体介导转入人高转移大细胞肺癌细胞株L9981中，反复传代培养，筛选出高效稳定表达荧光素酶的细胞株L9981-Luc。该细胞株能在体内外高效、持续稳定地表达荧光素酶基因；细胞株发光值和细胞数目呈直线线性关系（*R*^2^=0.973）；L9981-Luc细胞株在小鼠体内具有高成瘤和高转移特性，应用精诺真活体动物可见光成像系统（IVIS imaging system 200 Series）能直观地观察活体内原发瘤和转移瘤的情况；原发瘤重量和发光值呈直线线性关系（*R*^2^=0.900）^[2]^。IVIS系统具有高灵敏度，与传统方法相比，在肉眼尚不能发现肿瘤的时候即可使用该方法观察、量化活体内肿瘤细胞的生长，因此在早期即可开始对动物皮下自发转移模型进行药物处理。生物发光反映的是具有代谢活力细胞的数量，与通过测量肿瘤体积判断肿瘤细胞负荷的方法相比，避免了肿瘤周边水肿、肿瘤组织内坏死细胞和组织的干扰，更有效的反映了药物对肿瘤细胞生物学特性的影响。

硒是人类和哺乳动物必需的微量元素，许多流行病学资料和一些临床试验已初步证明超营养水平的硒摄入量对于包括食道癌、胃癌、结肠癌、肝癌、前列腺癌、卵巢癌及肺癌等各系统肿瘤的发生具有预防作用^[3-5]^。刘洁薇等^[6]^在体外应用甲基硒酸处理L9981细胞株，观察到甲基硒酸可明显抑制人高转移大细胞肺癌细胞株L9981的体外增殖和克隆形成能力，并诱导细胞凋亡；同时可明显上调*Fas*、*FasL*、*Bax*、*p21*、*p53*和*cyclinD1*基因表达水平。本研究观察到在活体动物体内，甲基硒酸对L9981-Luc细胞的原发瘤生长有明显的抑制作用，差异具有统计学意义，对甲基硒酸抗肿瘤作用的研究做了重要补充。本研究同时观察到在裸鼠体内，甲基硒酸具有抑制肺癌细胞株L9981-Luc移植瘤肺转移的趋势，但其确切机理有待进一步研究。通过各组裸鼠体重的观察比较，观察到50 μg甲基硒酸毒性小于顺铂的毒性作用，甲基硒酸组可较好的耐受，两者联合应用是否可以增加疗效有待进一步研究。

硒化合物抑制肿瘤生长及转移的机制可能与其抑制肿瘤血管生成、诱导细胞凋亡、抗肿瘤侵袭转移能力等有关^[7]^，有可能成为新的肺癌化疗的辅助治疗手段，有待于进一步研究。
